# Years of Life Lost Due to External Causes of Death in the Lodz Province, Poland

**DOI:** 10.1371/journal.pone.0096830

**Published:** 2014-05-08

**Authors:** Malgorzata Pikala, Marek Bryla, Pawel Bryla, Irena Maniecka-Bryla

**Affiliations:** 1 Department of Epidemiology and Biostatistics, the Chair of Social and Preventive Medicine of the Medical University of Lodz, Lodz, Poland; 2 Department of Social Medicine, the Chair of Social and Preventive Medicine of the Medical University of Lodz, Lodz, Poland; 3 Department of International Marketing and Retailing, University of Lodz, Lodz, Poland; Taipei Medical University, Taiwan

## Abstract

**Background:**

The aim of the study is the analysis of years of life lost due to external causes of death, particularly due to traffic accidents and suicides.

**Materials and Methods:**

The study material includes a database containing information gathered from 376,281 death certificates of inhabitants of the Lodz province who died between 1999 and 2010. The Lodz province is characterized by the highest mortality rates in Poland. The SEYLL_p_ (Standard Expected Years of Life Lost *per living person*) and the SEYLL_d_ (*per death*) indices were used to determine years of life lost. Joinpoint models were used to analyze time trends.

**Results:**

In 2010, deaths due to external causes constituted 6.0% of the total number of deaths. The standardized death rate (SDR) due to external causes was 110.0 per 100,000 males and was five times higher than for females (22.0 per 100,000 females). In 2010, the SEYLL_p_ due to external causes was 3746 per 100,000 males and 721 per 100,000 females. Among males, suicides and traffic accidents were the most common causes of death (the values of the SEYLL_p_ were: 1098 years and 887 years per 100,000 people, respectively). Among females, the SEYLL_p_ values were 183 years due to traffic accidents and 143 years due to suicides (per 100,000 people).

**Conclusions:**

A decrease in the number of years of life lost due to external causes is much higher among females. The authors observe that a growing number of suicides contribute to an increase in the value of the SEYLL_p_ index. This directly contributes to over-mortality of males due to external causes. The analysis of the years of life lost focuses on the social and economic aspects of premature mortality due to external causes.

## Introduction

The Lodz province is located in central Poland. It is inhabited by more than 2,500,000 people, which represents about 7% of the total population of the country. For many years, it has been characterized by the highest mortality rates for various causes. In 2010, the total mortality rate in Poland was 773.7, but in the Lodz province it was 860.0 per 100,000 people [1]. External causes of death are the third most common cause for both inhabitants of Poland and the Lodz region. The most common causes of death are cardiovascular diseases followed by malignant neoplasms [2]–[10]. An analysis of mortality in particular age groups confirms that external causes most often affect people aged 15–39 and they contribute to 45% of all deaths, which results in the greatest number of lost years [11]. In regard to external causes of death, traffic accidents and suicides contributed to almost half the number of deaths in all age groups of the Polish population: in 2010 the values were, respectively, 19.2% and 26.8% nationwide, and 18.8% and 25.1% in the Lodz region [12].

Some methods are becoming more valuable in evaluating the state of health, and thanks to these, it is possible to calculate the degree of premature mortality in terms of years of time lost. Time lost due to premature mortality is a function of the death rate and life expectancy. The analysis of the years of life lost focuses on the social and economic aspects of premature mortality. From the economic point of view, implementing preventative measures aimed at reducing the number of deaths contributing to the greatest number of lost years of life seems to be most effective.

A few methods can be used to measure the gap between the real death age in a particular population and the ideal death age, and the main difference between these methods is the point of reference used, i.e. the death age considered as “ideal”. *Murray* and *Lopez* suggest using the SEYLL method (*Standard Expected Years of Life Lost*) to measure the burden of disease due to premature mortality [13], [14]. The researchers suggest adopting the expected years of life in Japan as the standard life span because it is the longest. For Europe, the authors of “*Health statistics – Atlas on mortality in the European Union*” suggest adopting the life tables of 15 old European Union member states [15]. The application of life tables for all the 27 member states might be an alternative. Differences in the life span in the particular countries are still very high. For example, in 2010, the average life span for males from the European Union was 76.7 years. Inhabitants of Italy could expect the longest lifespan (79.8 years) and in Lithuania the lifespan was the shortest (68.0 years). In regard to females, the average life expectancy in 2010 in 27 countries of the European Union was 82.9 years. In Spain and France the life span was the longest (85.3 years) and in Bulgaria – the shortest (77.4 years) [1]. The application of the life tables for the whole European Union might cause a discrepancy in the number of years of life lost in the majority of the particular European countries in which the average life span is longer than that of the whole Union. This, in turn, could cause wrong conclusions. It is reasonable to compare the life expectancy in Central and Eastern European countries with the life expectancy in the 15 ‘old’ EU member states, because such a comparison clearly shows the gap between these two groups of countries, and is a motivating factor to catch up with the health standards which the 15 countries enjoy.

The Lodz region is characterized by the highest mortality rates due to external causes in comparison with the whole country. In 2010, the SDR in Poland was 93.2 per 100,000 males and 20.6 per 100,000 females [11]. In the Lodz province, the values were 110.1 per 100,000 males and 22.0 per 100,000 females.

The aim of this study is to analyze years of life lost by inhabitants of the Lodz province due to external reasons, including particularly traffic accidents and suicides.

## Materials and Methods

The research project was granted an approval of the Bioethics Committee of the Medical University of Lodz on 22 May 2012 No. RNN/422/12/KB. The study material includes a database which contains information gathered from 376,281 death certificates of inhabitants of the Lodz province, who died between 1 January 1999 and 31 December 2010, provided by the Regional Centre of Public Health in Lodz and the Department of Information of the Central Statistical Office for the purpose of this study In order to calculate the death rates, we used data on the size of population of the Lodz province on 30 June of the particular year [12]. To eliminate the influence of any age differences on the mortality rate and compare the time and location of deaths, the authors calculated SDRs according to the standard European population. The standardization procedure was carried out with the direct method.

In Poland, since 1 January 1997, death causes are coded according to the *International Statistical Classification of Diseases and Health Related Problems – Tenth Revision – ICD-10*. In that classification, external causes of morbidity and mortality (V01-Y98) include: transport accidents (V01-V99), other external causes of accidental injury (W00-X59), intentional self-harm (X60-X84), assault (X85-Y09), other (e.g. event of undetermined intent, legal intervention and operations of war, complications of medical and surgical care).

The SEYLL index (*Standard Expected Years of Life Lost*) is used to calculate the number of years of life lost by the studied population in comparison to the years lost by the referential (standard) population. Life tables of the 15 ‘old’ European Union member states were adopted as standard.

We should consider a population of size N and assume that d_xc_ stands for the number of deaths at the age of *x* due to a particular cause *c,* and 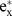 is the number of expected years of life that remain to be lived by the population which is at the age of *x*. If we assume that *l* is the last year of age until which the population lives, the number of years of life lost due to the cause *c* is calculated with the use of the following formula: 
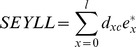



By dividing the absolute number of years lost due to cause *c*, calculated according to the following formula, by the number of deaths due to cause *c*, we obtain the average number of years of life lost by one person who died due to cause *c*.



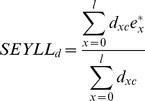



The authors also estimated the SEYLL_p_ indices determined by the number of the studied population [16]–[18].



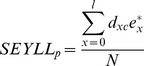



The analysis of time trends has been carried out with *joinpoint* models. The Joinpoint Regression Program is a statistical software package developed by the U.S. National Cancer Institute for the Surveillance, Epidemiology and End Results Program. The software takes trend data and fits the simplest joinpoint model that the data allow. The user supplies the minimum and maximum number of joinpoints. The program starts with the minimum number of joinpoint (e.g. 0 joinpoints, which is a straight line) and tests whether more joinpoints are statistically significant and must be added to the model (up to that maximum number). This enables the user to test that an apparent change in trend is statistically significant (p<0.05). The tests of significance use a Monte Carlo Permutation method. The software also allows to create graphs, where several different lines are connected together at the “joinpoints” [19], [20].

We have also calculated *annual percentage change* (APC) for the indices: SEYLL_p_ and SEYLL_d_ for each segment of broken lines with corresponding 95% *confidence intervals* (CI).

## Results

In the studied period, 24,294 deaths due to external causes were registered (table 1). The proportional mortality ratio (PMR) due to these reasons was 6.5%.

**Table 1:** The number of inhabitants, the number of all deaths and the number of deaths due to external causes divided into traffic accidents, suicides and other external causes by sex in the Lodz province from 1999 to 2010.

Year	Population	All deaths	Deaths due to external causes			
			Traffic accidents	Suicides	Other external causes	Total
**Males**						
1999	1,275,053	17,101	392	374	690	1456
2000	1,269,672	16,588	488	369	580	1437
2001	1,264,361	16,396	404	395	716	1515
2002	1,248,591	16,199	467	348	719	1534
2003	1,243,171	16,198	449	313	692	1454
2004	1,238,560	16,116	415	373	759	1547
2005	1,232,899	16,495	383	370	843	1596
2006	1,221,937	16,700	359	402	897	1658
2007	1,216,266	17,102	366	365	863	1594
2008	1,212,328	16,585	373	380	783	1536
2009	1,208,787	16,640	293	473	732	1498
2010	1,212,182	16,252	279	410	771	1460
**Females**						
1999	1,387,055	15,486	130	84	312	526
2000	1,382,241	15,040	144	78	303	525
2001	1,377,873	14,611	112	68	306	486
2002	1,367,685	14,276	143	72	281	496
2003	1,362,225	14,891	156	66	290	512
2004	1,358,056	14,389	127	63	301	491
2005	1,353,180	14,517	118	71	310	499
2006	1,344,261	14,643	118	67	336	521
2007	1,339,632	14,898	149	72	240	461
2008	1,336,533	14,913	139	83	330	552
2009	1,333,045	15,276	116	87	311	514
2010	1,330,254	14,969	76	63	287	426

Deaths caused by external factors registered in the Lodz region in 2010 made up 6.0% of the total number of deaths. This value was however quite different for different age and sex groups. External causes contributed to 9.0% of deaths among males and to 2.8% of deaths among females (table 2).

**Table 2:** Standardized death rates and percentage of deaths due to external reasons out of all deaths in the Lodz province from 1999 to 2010.

Years	Males		Females		Total	
	SDR (per 100,000)	Percentage	SDR (per 100,000)	Percentage	SDR (per 100,000)	Percentage
1999	112.7	8.5	30.4	3.4	69.8	6.1
2000	112.6	8.7	30.6	3.5	69.2	6.2
2001	118.0	9.2	28.4	3.3	70.9	6.5
2002	119.2	9.5	29.0	3.5	71.6	6.7
2003	112.7	9.0	29.8	3.4	69.2	6.3
2004	119.3	9.6	28.9	3.4	71.7	6.7
2005	122.9	9.7	29.0	3.4	73.6	6.8
2006	127.2	9.9	30.2	3.6	76.1	7.0
2007	121.1	9.3	26.5	3.1	70.7	6.4
2008	117.7	9.3	30.9	3.7	72.2	6.6
2009	114.4	9.0	27.4	3.4	68.5	6.3
2010	110.1	9.0	22.0	2.8	63.7	6.0

**Figure 1:**
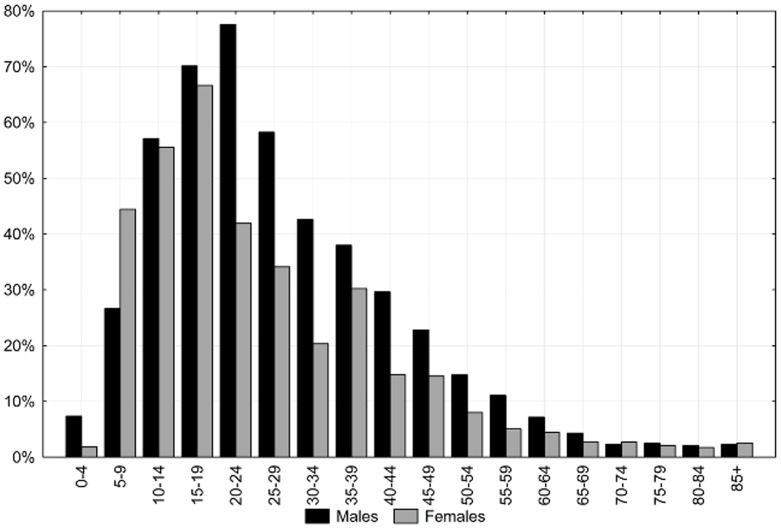
The proportion of deaths due to external causes by sex and age groups in the Lodz province in 2010.

The PMR due to external causes is very high among younger people, the highest among people aged 15–24 years. The older the person, the lower the value of the PMR and the decrease is secular (figure 1).

**Figure 2:**
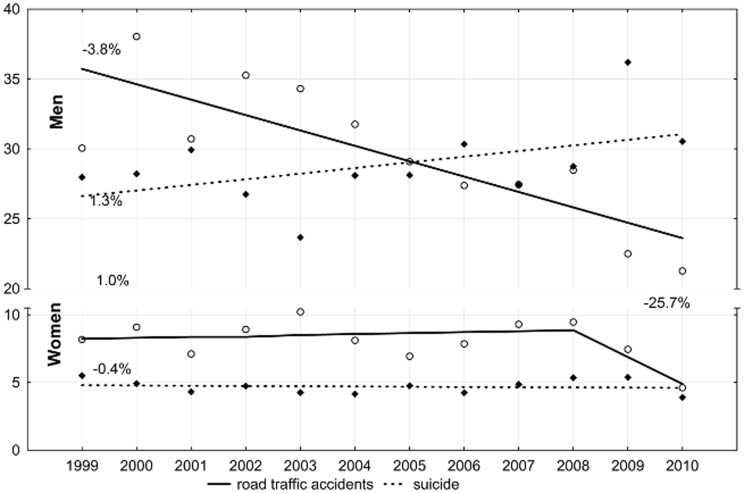
Time trends in standardized death rates due to road traffic accidents and suicide in Lodz province in 1999-2010 (per 100,000 persons).

The SDR due to external causes in 2010 was 110.1 per 100,000 males and this value was five times higher than for females (22.0 per 100,000 females). The male-to-female SDR ratio is increasing, as in 1999 it was 3.7 (112.7 vs 30.4). The cause of these increasing disproportions between males and females are differences in the direction and rate of the change in mortality trends. Changes in the mortality rate due to traffic accidents and suicides mostly contribute to changes in the SDRs due to external factors because the above causes contribute to almost half of deaths in the category of external causes. Among males, a decrease in the SDR was caused by a decrease in the mortality rate due to traffic accidents (it dropped annually by 3.8%; p<0.05). Suicide trends were completely different. An increase can be seen in the number of suicides among males between 1999 and 2010. The SDR value increased by 1.3% per year (p>0.05). Since 2006, the number of suicide victims has been higher than the number of victims of road accidents: the SDR values due to these two reasons being 30.5 and 21.3, respectively, per 100,000 males in 2010 (figure 2).

In regard to females, in the period 1999–2008, mortality due to traffic accidents increased on average by 1.0% per annum. After 2008, the SDR decreased dramatically (APC = −25.7%, p>0.05), and in 2010, it was 4.6 per 100,000 females. Little change was seen in female mortality due to suicides between 1999 and 2010 (APC = −0.4%, p>0.05). In 2010, the SDR due to this cause was 3.9 per 100,000 females. External factors, particularly traffic accidents and suicides, are the most serious life-threatening factors in the younger segments of the population and contribute to the following number of lost years. In 2010, the absolute SEYLL index was more than 45,000 years for males and more than 9,500 years for females. The SEYLL_p_ value was 3,746 standard expected years of life lost per 100,000 males and 721 per 100,000 females (table 3).

**Table 3:** Number of years of life lost due to external causes in absolute numbers (SEYLL), indices per 100,000 population (SEYLL_p_) and indices per 1 death due to these causes (SEYLL_d_) by sex in the Lodz province from 1999 to 2010.

Years	Males			Females		
	SEYLL	SEYLL_p_ (per 100,000)	SEYLL_d_	SEYLL	SEYLL_p_ (per 100,000)	SEYLL_d_
1999	46871	3676	32.2	12419	895	23.6
2000	44963	3541	31.3	13385	968	25.5
2001	47652	3769	31.5	12009	872	24.7
2002	48888	3915	31.6	12741	932	24.7
2003	46007	3701	32.1	13000	954	26.1
2004	48296	3899	31.4	12502	921	25.5
2005	50874	4126	31.2	12430	919	24.3
2006	52657	4309	31.8	13296	989	25.5
2007	49427	4064	31.0	12385	925	26.9
2008	49688	4099	32.3	14035	1050	25.4
2009	47609	3939	31.8	12597	945	24.5
2010	45418	3746	31.1	9593	721	22.5

Among males, suicides (SEYLL_p_ = 1,098 years per 100,000 males) and traffic accidents (SEYLL_p_ = 887 years per 100,000 males) contributed the most to the number of lost years of life. Among females, the SEYLL_p_ index in 2010 was 183 years due to traffic accidents and 143 years due to suicides per 100,000 females (table 4).

**Table 4:** Number of years of life lost due to external causes, traffic accidents and suicides in the Lodz province by sex in 2010.

		Males			Females	
Death causes	SEYLL	SEYLL_p_ (per 100,000)	SEYLL_d_	SEYLL	SEYLL_p_ (per 100,000)	SEYLL_d_
external causes	45418	3746	31.1	9593	721	22.5
including:						
traffic accidents	10750	887	38.5	2434	183	32.0
suicides	13307	1098	32.5	1909	143	30.3

Among males, the trend of lost years due to external causes was increasing from 1999 to 2006 (APC = 2.4%, p<0.05). After 2006, the SEYLL_p_ index started to decrease at the annual rate of 2.4% (p>0.05) (table 5).

**Table 5:** Time trends of the SEYLL_p_ by sex in the Lodz province from 1999 to 2010 – joinpoint regression analysis.

Death causes	Joinpoints	Period	APC	95% CI	
**Males**					
external causes of death	1	1999–2006	2.4*	−1.0	3.8
including:		2006–2010	−2.4	−5.5	0.8
traffic accidents	0	1999–2010	−2.8*	−4.4	−1.1
suicides	0	1999–2010	1.7	−0.2	3.6
**Females**					
external causes of death	1	1999–2008	1.3*	0.1	2.5
including:		2008–2010	−13.2*	−23.8	−1.2
traffic accidents	1	1999–2008	1.5	−3.3	6.5
		2008–2010	−26.0	−56.4	25.7
suicides	0	1999–2010	−0.4	−2.7	2.0

* p<0.05

**Figure 3:**
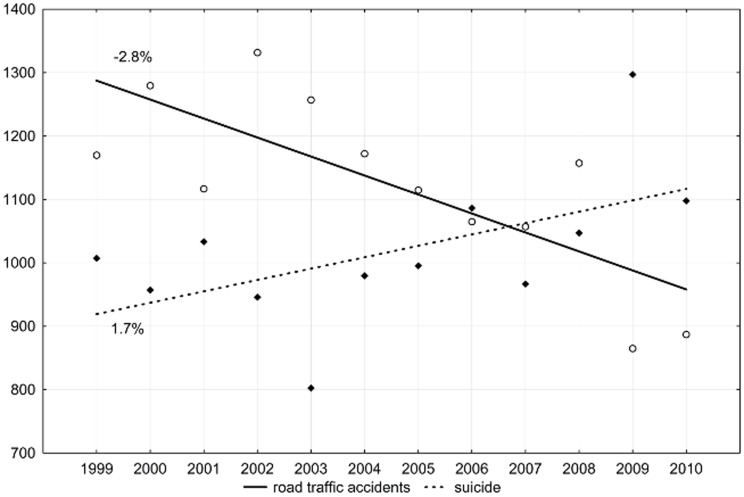
Time trends in SEYLL_p_ of males due to road traffic accidents in Lodz province in 1999–2010 (per 100,000 males).

The authors observed a reverse trend of years of life lost due to traffic accidents and suicide among males. The value of the SEYLL_p_ index was growing due to suicides by 1.7% (p>0.05) per year. The increase was accompanied by a decrease in the number of years lost by males due to traffic accidents (APC = −2.8%, p<0.05) (figure 3).

**Figure 4:**
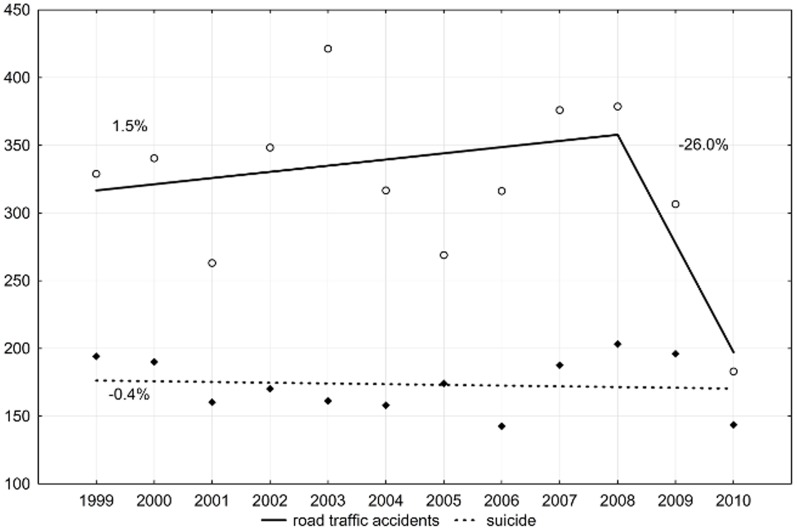
Time trends in SEYLL_p_ of females due to road traffic accidents in Lodz province in 1999–2010 (per 100,000 females).

Among females, the growing trend of the number of lost years of life due to external causes changed in 2008. This change was influenced by deaths due to traffic accidents. In the period 1999–2008, the SEYLL_p_ index was growing by 1.5% per year (p>0.05) and after 2008, it started to decrease by 26% per year (p>0.05). The number of years of life lost among females due to suicides changed only slightly in the whole period under study (APC = −0.4%, p>0.05) (figure 4).

The SEYLL_d_ index, which is identified as the ratio of the number of lost years of life due to external reasons and the number of deaths due to these reasons, shows that a male who died in 2010 lost on average more than 31 years of life and a female lost more than 22 years. The SEYLL_d_ indices were even higher for traffic accidents and suicides. The values were respectively: 38.5 years and 32.5 years for one dead male and 32.0 and 30.3 for one dead female. It should be pointed out that the number of lost years of life for one person who died in a traffic accident or committed a suicide is the highest of all reasons for death. The values of the SEYLL_d_ indices in the period 1999–2010 fluctuated slightly. The indices were changing dynamically because of traffic accidents which affected males. The annual increase was 1% (p<0.05) (table 6).

**Table 6:** Time trends of the SEYLL_d_ by sex in the Lodz province in 1999–2010 – joinpoint regression analysis.

Death causes	Joinpoints	Period	APC	95% CI	
**Males**					
external causes of death	0	1999–2010	−0.1	−0.3	0.2
including:					
traffic accidents	0	1999–2010	1.0*	0.5	1.4
suicides	0	1999–2010	−0.1	−0.5	0.2
**Females**					
external causes of death	1	1999–2008	0.7	−0.3	1.7
including:		2008–2010	−6.8	−16.6	4.2
traffic accidents	0	1999–2010	0.3	−0.8	1.3
suicides	0	1999–2010	−0.5	−1.6	0.6

* p<0.05

## Discussion

As the given numbers refer to all the inhabitants of the Lodz region who died in a twelve-year period, our research study can be regarded as quite exhaustive. The year 1999 was chosen as the beginning of the study period because at that time, the Polish authorities introduced administrative reforms which reorganised the boundaries of regions. Any comparative analyses encompassing the period prior to the new administrative division are not feasible.

It is worth noting that deaths caused by malignant neoplasms contributed to a loss of 16.5 years per male and 18.4 years per female [21]. In the group of cardiovascular diseases, which contribute the most to the absolute number of lost years of life, the SEYLL_d_ indices were even lower – 13.9 years for one dead male and about 11.8 for one dead female [22].

The analysis of changes in mortality of inhabitants of the Lodz region due to external reasons confirms negative trends in the mortality rate which is caused by male suicides. A number of studies indicate that the mortality rate due to suicide depends on the geographical location, climate (including the intensiveness of exposure to the sun), cultural factors (including religion), various occurrence of mental diseases [23] and social and economic conditions [24]–[28]. In Europe, the greatest number of suicides is observed in Finland, Hungary and the Baltic states; inhabitants of countries located in the south of Europe are least prone to commit suicide [29]–[32].

Political and constitutional changes which took place in former communist countries of Central Europe [33], [34] also contributed to the number of suicide attempts. In Poland, the death rate due to suicide increased between 1970 and 2009 from 11.2 to 17.0 (by 51%) [35]. Such a significant increase is highly contrastive to trends observed in other European countries, in which the number of suicides started to drop at the beginning of the 1990s. Although the increase is thought to be great, precise government statistics concerning suicide attempts were hard to obtain during the socialist period, and no study has ever confirmed the reliability of data registered before 1989 in Poland. An increase in the standardised mortality rates due to suicides among males contributes to a rising mortality trend due to these reasons. Among females, the value of the mortality rate remained at a similar level between 1970 and 2010. This trend results in the highest male-to-female suicide ratio in Europe, i.e. 7∶1 (in the Lodz province 5∶1). One of causes of such great differences might be the fact that women, more often than men, use so-called “soft” suicide methods, such as poisoning or drowning. This could lead to a wrong classification of the cause of death [36]–[37]. Men are more prone to commit suicide and to be affected by “male depression” [38]. Men more often than women suffer from stress, become addicted to alcohol and less frequently ask for help. Such problems are particularly visible in central European countries, where social and economic changes as well as increased unemployment lead to fewer opportunities for males and to their social exclusion [39], [40]. Women are not affected by the above negative social phenomena to such an extent as men. They are protected by maternity duties, use social help more often than males and are less prone to risk-taking and becoming addicted to alcohol [41].

Despite a decrease in the number of deaths due to traffic accidents in Poland, the death rate is still one of the highest in Europe. In 2010, a higher mortality rate was observed only in Romania, Greece and Latvia [1]. In the Lodz region, this situation is even more serious. Mortality due to traffic accidents was 21.3 per 100,000 males and 4.6 per 100,000 females and was higher than the mean value for Poland (17.9 and 4.5 respectively per 100,000 males and females). The authors of *Health in the European Union* point out that between 1985 and 2002 the number of victims of road accidents in the EU-15 (15 member states of the European Union before the eastern enlargement in 2004) dropped by 36% whereas in the EU-10 (10 countries, mostly from Central and Eastern Europe, that joined the European Union in 2004) it increased. In 2002, Poland, Slovakia, Latvia, Estonia and the Czech Republic observed higher mortality rates due to these reasons compared to 1985 [42]. The bad condition of roads, poor technical condition of vehicles and reckless driving, especially exceeding speed limits and driving under the influence of alcohol, contribute to a great number of traffic victims [43], [44]. Databases of the Main Headquarters of Police are the most reliable source of information on traffic accidents in Poland. None of the causes listed by the police concerns poor quality of roads, as it is difficult to determine. There is no doubt, however, that the condition of Polish roads gets improving. According to an assessment of the technical condition of the surface of national roads, performed by the Directorate-General for National Roads and Highways in Poland at the end of 2012, 64% of the national roads are in a satisfactory condition [45]. It is possible to identify the number of victims of traffic accidents caused by drivers under the influence of alcohol. In 2011, road users under the influence of alcohol took part in 12.1% of traffic accidents and they accounted for 14.6% of all deaths in traffic accidents [46]. This share declines systematically. Compared to 2001, the number of accidents with the participation of persons under the influence of alcohol dropped by as much as 33.1%. The largest group of drunk accident makers consisted of drivers. Out of all accidents caused by drivers, 8.4% were attributed to drivers under the influence of alcohol.

Traffic accidents are the most common cause of death among people under 25 years of age [13], [47]. According to the WHO, 75% of people involved in traffic accidents are men. The difference is even more visible among people aged 15–29, where males make up 80% of the total number of victims. It results from the fact that the majority of drivers are males and they more often use vehicles which are involved in accidents, e.g. motorbikes. They also exceed speed limits more often than women and drive under the influence of alcohol [48], [49].

People aged 15–39 are at a high risk of death due to external causes. External causes contribute to the greatest number of years of life lost in this age group. In countries where external causes contribute to the lowest number of lost years of life (England, Holland, Germany, Spain, Italy), the average value of the SEYLL_p_ is between 1000 and 1400 years per 100,000 males and between 100 and 600 years per 100,000 females. In countries where external causes contribute to the highest number of lost years of life (countries of Central and Eastern Europe, Finland, Portugal and France), the average value of the SEYLL_p_ is between 2900 and 8700 years per 100,000 males and between 1000 and 2300 years per 100,000 females. Traffic accidents contribute to the highest number of years of life lost due to external reasons. In countries where the mortality rate due to these causes is high, the average value of the SEYLL_p_ is between 1200 and 2200 per 100,000 males and between 300 and 500 per 100,000 females, and is five times higher than in low mortality countries. In high mortality countries, suicides contribute to a loss of 1200 years of life per 100,000 males and 300 years per 100,000 females. In countries which are characterized with low mortality, the loss is four times lower, and the values are 300 years per 100,000 males and 80 years per 100,000 females [9]. The above data confirm that the Lodz region is characterized by a great loss of years of life lost due to external causes among both male and female inhabitants. Of these causes, suicides contribute to the greatest number of years of life lost among males, and traffic accidents cause the greatest losses among females.

### Limitations of the study

We are well aware of the limitations of the present study. One of these limitations was the 12-year duration of the study period, which was not particularly long in the context of evaluation of trends regarding the SEYLL. However, extending this period to years before the major administrative reform in Poland that took place on January 1, 1999 would introduce a significant bias related to changes in the area, and to the population of the Lodz region. On the other hand, the 12-year study period was long enough to introduce some variation in the quality of reporting and registration of causes of death.

We believe, however, that the present analysis of data from more than 375,000 death certificates, including 24,325 deaths due to external causes, and the use of methodology to evaluate life years lost addresses the gap in the Polish literature regarding economic and social losses due to these diseases, and allows international comparisons of our data.

The reliability of statistical analysis on the basis of deaths depends to the largest extent on the correct identification of the primary death cause, in particular among the elderly. Taking that in consideration, certain changes were introduced in Poland in 2009. In order to standardize death causes, which are subject to further statistical analyses, it was determined that the doctor who states the death is responsible for filling in the death card, into which he or she puts the primary, secondary and direct death cause, whereas qualified teams of doctors are responsible for coding death causes according to the ICD-10 classification. The duties of a dozen of regional statistical offices were taken over by the Polish Central Statistical Office. The relatively short time that new system of processing data on deaths has been operating impedes its evaluation.

## Conclusions

The number of years of life lost due to all external causes in the Lodz region increased among males in the period 1999–2006, and among females from 1999 to2008. The SEYLL index has been decreasing among males since 2006 and since 2008 among females. The index is decreasing much faster among females than among males.

The value of the SEYLL_p_ index due to suicides among males increased during the period 1999–2010. Since 2006, the number of deaths due to suicide among males has been higher than the number of traffic victims.

Traffic accidents and suicides are the most common causes of loss of years of life per a dead person.

There is a need to design and implement psychological programmes aimed at young males, to enhance their ability to cope with difficult situations and actively seek employment.
